# An Automated Hybrid Deep Learning-based Model for Breast Cancer Detection using Mammographic Images

**DOI:** 10.2174/0115734056427227251219161545

**Published:** 2026-02-19

**Authors:** Ongole Gandhi, S. N. Tirumala Rao, M. H. M. Krishna Prasad

**Affiliations:** 1 Department of Computer Science & Engineering (CSE), Jawaharlal Nehru Technological University, Kakinada, India; 2 Department of Computer Science & Engineering (CSE), Narasaraopeta Engineering College, Narasaraopet, Andhra Pradesh, India

**Keywords:** Breast cancer, Breast tumor, Classification, Deep learning, Feature extraction, Feature optimization, Mammographic images

## Abstract

**Introduction:**

Breast cancer is a disease in which abnormal breast cells grow uncontrollably and develop into a tumor. It is one of the most common cancers that affects women around the world. The most often used imaging tool for identifying breast cancer is mammography. Early and accurate identification of tumors can be crucial for effective treatment and planning, which reduces mortality rates. This work proposes a novel hybrid deep learning-based automated framework for early and accurate breast cancer detection using mammographic images.

**Methods:**

The methodology integrates multiple components into a hybrid model. Initially, mammographic images from the Curated Breast Imaging Subset of the Digital Database for Screening Mammography (CBIS-DDSM) undergo a preprocessing step, uses a guided filter to remove noise and enhance the visibility of regions of interest (ROI). Modified Dingo Optimization (MDO) algorithm is used to segment the tumour-affected regions, not only to identify abnormalities localized in a single region of the breast but also to effectively detect multiple abnormal areas distributed across different tissue regions. Deep features are then extracted using a pretrained U-Net architecture. The Search and Rescue Optimization (SRO) algorithm was utilized for feature optimization to select the most relevant deep features, reducing dimensionality and enhancing the model's diagnostic accuracy. A Dual Stage Spiking Convolutional Neural Network (DSS-CNN) is implemented for classification and enhancing the model's ability.

**Results:**

The proposed hybrid deep learning model achieves outstanding performance, with an accuracy of 98.598%, precision of 97.343%, recall of 97.514%, and an F-measure of 96.89%. Comparative analysis confirms that the approach significantly reduces false positive and false negative rates, outperforming existing state-of-the-art techniques.

**Conclusion:**

The proposed robust end-to-end system for early and accurate breast cancer detection demonstrates the efficacy of the framework in improving diagnostic accuracy, precision, recall, and F-measure, offering valuable support in clinical decision-making.

## 1 INTRODUCTION

Early detection of breast cancer is critically important for improving patient survival and clinical outcomes. When detected early, breast cancer can often be successfully treated withtargetedtherapies,reducingtheneedformoreseveretreatments like chemotherapy or surgery. According to studies, the five-year survival rate exceeds 90% when breast cancer is detected before metastases, whereas late-stage detection significantly reduces survival chances. As a result, an early and accurate diagnosis is critical for reducing mortality and improving the patient's quality of life.

Mammography, the most widely used imaging technique in both developed and developing countries, is an effective method for breast cancer screening. It provides a non-invasive, cost-effective method for detecting minute tissue abnormalities and microcalcifications that would otherwise go undetected during a physical examination. Regular mammography scree-ning detects tumors in their early stages, often years before clinical symptoms appear. Mammography also assists doctors in planning treatments and tracking the progression of illness.

While mammography has reduced breast cancer deaths, it does have limitations. Challenges such as overlapping breast tissues, differences in breast density, and radiologist subjectivity can all lead to inaccurate results, causing patient anxiety and diagnostic uncertainty. Using deep learning in mammography can improve image interpretation accuracy, reduce diagnostic errors, and lead to more reliable early detection results.

Breast tumors account for 25% of all tumor cases found in women in both developed and developing countries, according to the WHO [1, 2]. In recent decades, early detection and treatment methods have greatly improved survival rates, emphasizing the necessity of early diagnosis. Breast cancer diagnosis has traditionally used physical examination, imaging, and tissue biopsy [3]. Mammography, a low-dose X-ray imaging innovation for breast tissue from bosom, and it is widely utilized and effective in bringing down bosom cancer mortality; however, it can cause misleading results, negatives, and uneasiness [4]. Artificial intelligence (AI) procedures, machine learning (ML), and deep learning (DL) have changed clinical picture handling, including mammography translation for bosom malignant growth screening [5]. DL calculations can assist radiologists with spotting abnormalities early and reducing interpretation time [6]. To incorporate these cutting-edge innovations into clinical practice, simulated intelligence models should be vigorous and generalizable across assorted populations and medical care settings, and moral and administrative issues should be tended to [7].

Typically used to evaluate palpable masses or identify solid and cystic lesions on mammography, breast ultrasound uses sound waves to create images of the breast matter [8]. X-ray gives thorough breast tissue pictures with high responsiveness, however, lower particularity than mammography, and may increase misleading up-sides [9]. Biopsy strategies can involve percutaneous testing of breast tissue utilizing center needle biopsy or vacuum-assisted biopsy, or careful evacuation for obsessive examination [10]. Convolutional neural network (CNN), recurrent neural network (RNN), and additional deep learning designs have been used to develop algorithms that can outperform CAD systems and human experts in certain diagnostic tasks [11, 12]. DL-based breast cancer detection methods can extract complicated hierarchical features from raw mammogram images without feature engineering or segmentation [13]. Traditional CAD systems use handcrafted features and heuristics. DL procedures may also study and recover from new numbers, making them ideal for dynamic healthcare contexts with growing diagnostic databases [14].

DL-based breast cancer screening methods show promise, but their widespread use in clinical practice needs overcoming many issues [15].

Breast cancer screening programs worldwide rely on mammogram images for early detection and diagnosis [16]. Mammography is also non-invasive, cost-effective, and easy to conduct in outpatient settings, making it accessible to many patients and demographics [17]. DL models' interpretability and transparency are major issues, especially in medical settings where diagnostic predictions are crucial to decision-making [18]. ML/DL techniques in breast cancer diagnosis have great potential to improve medical imaging and patient care, despite the obstacles. DL algorithms can help radiologists’s anomalies early, reduce interpretation time, and improve diagnosis accuracy by automating mammography interpretation [19]. An AI-driven decision support system helps the doctors provide more precise, efficient, and patient-centered risk assessment [20]. Multidisciplinary teams of physicians, data scientists, and policymakers will be needed to fully utilize ML/DL in breast cancer screening and management. Automated DL techniques offer a promising approach for developing clinical tools that can reduce false positive and false negative results in mammography screenings. These techniques are effective at achieving high accuracy in analyzing heterogeneous mammographic images. DL has shown significant advancements in disease detection and diagnosis.

### 1.1 Contributions

An early and accurate hybrid deep learning model is proposed for breast cancer detection using mammograms. The major contributions of the proposed model are given as follows.

(1) The MDO algorithm is used to segment the ROI in mammograms.

(2) U-Net pretrained architecture used for feature extraction.

(3) The SRO algorithm is applied to optimize the features extracted from the mammograms.

(4) Employ DSS-CNN for the ultimate identification and categorization of breast cancer.

(5) The performance of the model is validated using the publicly available CBIS-DDSM dataset.

### 1.2 Related Works

This part contains a review of the literature on the use of ML/DL approaches for breast cancer detection and classification. More complex DL models and optimization methods are clearly on the rise, according to the research, which will result in breast cancer detection systems that are more accurate and dependable. The summary of research gaps derived from the current state-of-the-art efforts is presented in Table **1**.

### 1.3 State of the Art Works

Sahu *et al*. 2024 [21] introduced a breast cancer-specific DL based ensemble classifier. An efficient strategy improves the accuracy with limited datasets by integrating three effectual transmission learning models. Outstanding learning, depth-wise divisible intricacy, and upturned lingering blockage edifice improve speed and optimization, while Laplacian of Gaussian and adapted high-boosting advance performance. On the mini-DDSM dataset, the system detected abnormality and malignancy with 99.17% and 97.75% accuracy, respectively. The ultrasound datasets (BUSI and BUS2) had 96.92% to 97.50% accuracy.

Kalpana *et al*. 2024 [22] developed a computer learning-based mammography image feature extraction, categorization, and optimization for breast cancer prediction. After noise removal and normalization, probabilistic analysis of principal components is used to extract features from mammography images to detect tumors. The tumor location is classified using a Naïve Bayes classifier and a convolutional neural network that contains transmission. The optimization is done using the firefly binary grey and metaheuristic moth flame lion. Experiments conducted on different datasets demonstrate the effectiveness of the framework. On the INbreast dataset, the ensemble model that used the Bayes + FBGO classifier and TCNN+MMFLO optimizer achieved 95% and 98% accuracy rates, respectively.

Atrey *et al*. 2024 [23] presented the first semi-automated multimodal breast tumor classification system using mammography and ultrasound information. Forty-two gray-scale structures are retrieved, and numerical examination determines the best ones for tumor classification. A fresh database of mammograms and ultrasounds, enriched with various data augmentation and filtered to reduce artifacts and noise, is used to assess the proposed multimodal approach's performance with multiple machine learning classifiers. The results reveal that the cubic support vector machine (SVM) improved classification accuracy to 98.84% from 93.41% and 91.67% for mammography and ultrasonography alone.

Chowdhury *et al*. 2024 [24] have introduced a DL based breast cancer diagnosis system that uses a convolutional neural network (CNN) to automatically analyze mammogram images to help radiologists diagnose faster and more accurately. To improve image quality, a state-of-the-art CNN architecture is pretrained on a large dataset, followed by rigorous training, validation, and fine-tuning to ensure robustness and generalizability. Data augmentation and transfer learning boost the model's accuracy, outperforming classic mammography analysis approaches. The approach has great specificity in eliminating false positives and sensitivity in recognizing true positives, suggesting it could significantly improve breast cancer detection rates.

Shibu *et al*. 2024 [25] announced a quantum spinal net (Q-SpinalNet), a mammography-based breast cancer detection method. Preprocessing mammography images with the non-local means filter improves image quality. Edge-attention guidance and segmentation Network depends on the random variable constant used to segment. Q-Spinal Net detects BC using DQNN and Spinal Net. Q-Spinal Net had 90.3% accuracy, 90.9% TNR, and 90% TPR, indicating its promise as a breast cancer screening tool.

Mahmood *et al*. 2024 [26] proposed DL for breast lesion discovery, localization, risk valuation, and organization to reduce false positives and increase convergence rates. Chaotic leader selective filler swarm optimization (cLSFSO) extracts textural and statistical information to identify breast-dense lesions. Cancerous polyps are diagnosed and graded using CNN+LSTM and CNN+SVM hybrid DNN methods from pre-segmented ROIs. Transfer learning speeds breast mass classification and computing. Grad-CAM improves breast anomaly diagnosis. With standard and secluded databases, the procedures have compassion of 0.99 and an overall AUC of 0.99, representative important mammography examination advances.

Tariq *et al*. 2024 [27] presented a multi-level strategy for finding cancerous spots by using the grasshopper optimization algorithm, optimization CNNs for image segmentation, and image noise reduction. The GRASP algorithm improves feature extraction and selection optimization precision and computational efficiency. This method improves breast cancer diagnostic accuracy *via* CNN-based max segmentation, image noise reduction, and unique grasshopper optimization. A fuzzy grasshopper optimization algorithm improves feature extraction and selection, making the method unique, which achieves sensitivity 75%, specificity 97%, PPV 99%, NPV 45%, and accuracy 96%, making it a promising breast cancer diagnostics advancement.

Kanya *et al*. 2024 [28] have proposed the four-stage procedure for improving classification performance through feature selection: pre-processing, feature extraction using advanced gray-level co-occurrence matrix (AGLCM) and contrast-limited histogram equalization (CLAHE), choosing features using WA-BTLBO based on classification accuracy, and classification using an XGBoost classifier. This study compares many neural network architectures utilizing publicly available mammography images from the MIAS dataset, including SVM, RF, ANN, and KNN. Helping physiologists and radiologists detect breast cancer and potentially extending patient lifespan, WA-BTLBO with XGBoost classifier surpasses previous feature selection approaches and state-of-the-art methodologies. Plans include implementing metaheuristic approaches for use with bigger databases and a variety of disorders.

Qian *et al*. 2024 [29] presented ZFNet-based mammography image analysis for a typical breast cancer detection. Wiener and CALHE filters validate preprocessing processes, and then a trained ZFNet is adapted and qualified on the CBIS-DDSM database. An extreme learning machine (ELM) replaces leftover layers, using an optimum technique to calculate the appropriate number. An upgraded chimp optimization algorithm (SWChOA) and meta-heuristic optimization improve the classification performance. Through 10 hold-out validations, ZFNet-SWChOA-ELM shows superior performance, suggesting it could be used to diagnose breast cancer.

Boudouh *et al*. 2024 [30] proposed a comprehensive mammography pre-processing approach and tumor identification algorithm to improve cancer detection. Pre-processing used filters, whereas tumor detection used deep learning methods like transfer learning, data augmentation, and global pooling. The CNN architecture for mammography classification used six pre-trained CNN models for feature extraction and global pooling for classification. Well-selected pre-processing filters let InceptionResNetV2 and VGG16 models achieve 96.42% accuracy.

Akherati, Hossein, *et al*. 2025 [31] presented a model-free sliding mode attitude controller and observer framework for uncertain space systems, compensating for unknown dynamics and disturbances through a time delay estimation method instead of depending on an explicit system model. The design ensures finite-time stability of closed-loop attitude control and state-estimation errors in the presence of uncertainties by combining time-delay estimation with sliding-mode observer/control techniques to ensure finite-time convergence.

Ahmadi, *et al*. 2025 [32] used a Random Forest-based classifier to predict treatment response in Ulcerative Colitis (UC) patients and identified key biomarkers that improve classification performance. Integrating biomarker identification with predictive modeling allows for personalized treatment decision support. The classifier can categorize UC patients as likely responders or non-responders before therapy initiation.

Razavi, *et al*. 2025 [33] study presented, EEG analyses of Alzheimer's patients reveal clear abnormalities in brain-state transition dynamics, which may indicate altered large-scale neural network flexibility in the illness. The idea that brain-state dynamic metrics may function as biomarkers of network degeneration is supported by the discovery that these altered transition patterns, such as changes in dwell times in particular states and changes in how frequently one state transitions to another, correlate with cognitive performance/decline in Alzheimer's patients.

Najafi, *et al*. 2024 [34] proposed a three-step deep-learning pipeline for 3D lung-tumor reconstruction from CT scans, that combines: (i) a GAN with reinforcement-learning to segment lung tissue; (ii) a second GAN with a novel loss function to detect tumors; and (iii) a VGG16 feature-extractor paired with an LSTM and a GAN (with dilated-convolution discriminator) to generate the 3D tumor geometry. On the public LIDCIDRI dataset, they show significant improvements in performance. For instance, they achieved a lung segmentation IoU = 0.972 (compared to ~0.762 for U-Net) and an HD (Hausdorff Distance) of 0.925; for 3D reconstruction, they reported HD = 0.986 and Euclidean Distance = 1.126, outperforming multiple baseline methods.

Mahdavi 2022 [35] presented a novel hybrid architecture in order to simultaneously detect and segment brain tumors at the pixel level in MRI images, which combines the well-known U-Net segmentation network with a convolutional neural network classifier. The model outperformed many previous methods in this dataset context, with a reported accuracy of ~98% on the test set using the publicly available BRATS2020 dataset (1840 MRI images of size 240×240).

### 1.4 Recent Developments in AI and Deep Learning for Cancer and Tumor Detection

Recent advances in artificial intelligence and deep learning have significantly improved cancer and tumor detection by employing powerful models such as Vision Transformers, Swin Transformers, and foundation models like SAM and CLIP. These models use self-attention mechanisms to improve tumor localization and segmentation across multiple imaging modalities. Deep learning frameworks now incorporate multimodal data (radiological, pathological, and genomic) to make precise radiogenomic predictions for personalized treatment. Furthermore, self-supervised and federated learning approaches address data scarcity and privacy concerns, allowing models to train on decentralized datasets while keeping patient information private. Generative and explainable AI (XAI) techniques such as GANs, diffusion models, Grad-CAM, and SHAP improve dataset diversity, model interpretability, and clinician confidence in AI-powered diagnostics. Real-time and edge-based detection systems are revolutionizing cancer screening in low-resource settings.

Furthermore, real-time and edge-based detection systems are revolutionizing cancer screening in low-resource settings, enabling mobile and portable imaging solutions for early detection. Hybrid architectures such as Attention U-Net, DenseNet-U-Net, and Transformer-CNN models improve segmentation and classification accuracy by capturing complex structural and contextual details. AI-assisted diagnostic systems are increasingly being clinically validated, with several receiving FDA approval for integration into screening workflows. The current focus is also on developing ethically sound and bias-aware AI systems that ensure fairness, transparency, and data security, thereby boosting trust and reliability in clinical decision-making.

However, there are still problems with the current state-of-the-art models.

Clinical acceptance is hampered by deep learning models' limited interpretability and transparency. When models are trained on small or unbalanced datasets, overfitting and poor generalization happen. Efficiency and diagnostic speed are impacted by feature redundancy and high computational costs. Tumor regions are not fully localized due to inadequate segmentation accuracy, which lowers classification reliability.

Although effective, current methods of breast cancer detection suffer from some limitations that limit their clinical utility and diagnostic efficiency. The traditional diagnosis methods, such as thresholding, doubling thresholds, clustering, and region growth, are unable to detect the irregular.

### 1.5 Problem Description and Definition

The motivation for this research stems from the urgent need to improve the accuracy and reliability of breast cancer detection. Current methods often fall short in providing consistent results across diverse populations and varied imaging conditions. Automated detection systems powered by DL have shown significant promise in enhancing diagnostic accuracy. However, existing DL models need to be further refined to handle the heterogeneity of mammographic images effectively. Mohiyuddin *et al*. [36] proposed a modified YOLOv5 network for detecting and classifying breast tumors. The preprocessing step incorporates picture improvement methods and the removal of pectoral muscles and marks. The examination was conducted with bunch size of 8, a learning rate of 0.01, an energy of 0.843, and an age of 300. The outcomes show that the proposed model beats YOLOv3 and Quicker R-CNN, accomplishing a mean average precision (mAP) of 96%, an MCC value of 93.50%, an accuracy of 96.50%, a false positive rate (FPR) of 0.04, and a false negative rate (FNR) of 0.03. The high rate, which can cause patients unnecessary anxiety and result in missed diagnoses, is one of the most significant obstacles to automated mammogram analysis. Detection accuracy suffers as a result of traditional methods' inability to effectively isolate these areas.

The proposed work modified dingo optimization (MDO) for fragmenting returns for capital invested in mammograms. It is powerful in disengaging growth sores, which improves the element extraction process. By precisely portioning the returns for capital invested, the model guarantees that the most applicable regions are broken down, further developing the general location execution. Separating and upgrading the right highlights from mammographic pictures is essential for exact characterization. Many existing strategies neglect to improve includes successfully, resulting in below-average execution. To address this, we use the U-Net to extract profound elements from mammograms and improve the location exactness by guaranteeing that excellent, significant highlights are captured. The search and rescue optimization (SRO) is utilized for including improvement, choosing the most ideal elements, and decreasing information dimensionality. Many existing models are either excessively shortsighted or not powerful enough to deal with the intricacy of mammographic information, bringing about lower accuracy rates. Our dual-stage spiking convolutional neural network (DSS-CNN) is utilized to deal with the intricacy of mammographic pictures. By utilizing a two-stage process, the DSS-CNN really catches and cycles many-sided designs within the information, prompting further developed characterization exactness. Our structure is utilized to deal with enormous and heterogeneous datasets effectively. The mix of the U-Net pretrained design and the SRO calculation guarantees that the model can process and enhance highlights from different information sources. This ability permits our model to keep up with elite execution across shifted mammographic pictures, guarantee the dependable identification and grouping paying little mind to information heterogeneity. We have formulated the following results objectives to address those issues.

(1) Ensure effective isolation of tumor lesions to enhance the feature extraction process, improving overall detection accuracy.

(2) Capture high-quality, relevant features and reduce data dimensionality to improve the classification accuracy.

(3) Enhance the accuracy of early breast cancer detection by identifying and analyzing complex patterns in the data.

(4) Maintain high performance and reliable classification across varied mammographic images, addressing the challenges posed by data diversity and volume.

## 2 METHODOLOGY

**Fig. (1):**
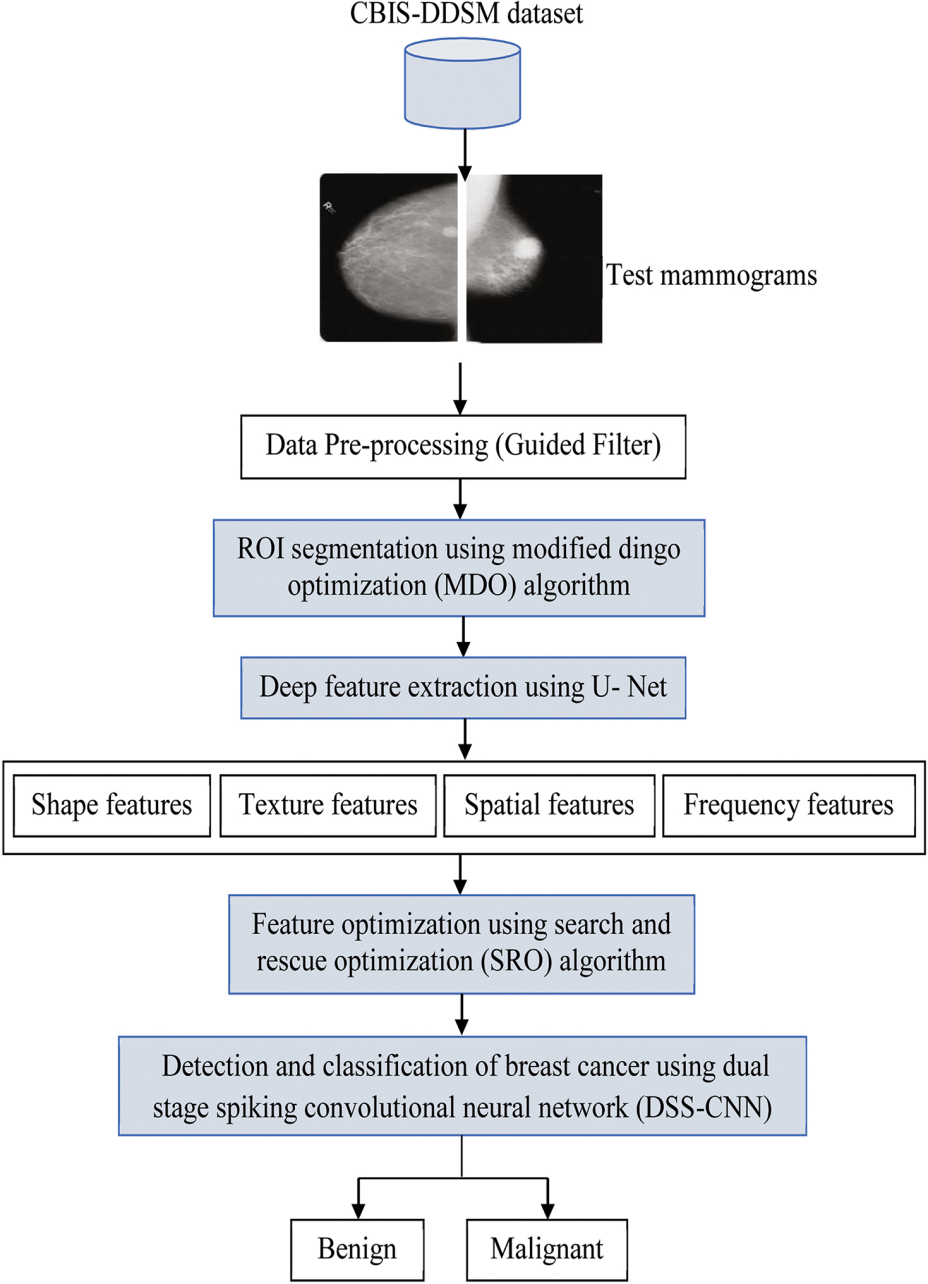
Proposed model for a hybrid DL-based classifier and feature optimization for early breast cancer detection using mammograms.

Figure **1** shows the proposed model intended for the early discovery of bosom malignant growth utilizing mammograms, utilizing a combination of element enhancement and a half and half profound learning classifier. The interaction starts with the choice of the CBIS-DDSM dataset, an openly accessible asset containing mammogram pictures marked with harmless and dangerous classes. These pictures act as the establishment for preparing and testing the breast cancer discovery model. Preprocessing is the next step after getting mammogram images from the dataset. The modified dingo optimization (MDO) calculation is utilized to portion areas of interest (returns for capital invested) inside the mammograms. When the returns on initial capital investment are distinguished, profound component extraction is performed utilizing the U-Net design. This interaction includes removing different kinds of elements, including shape highlights, surface highlights, spatial elements, and recurrence highlights, from the sectioned returns for money invested. Following element extraction, the search and rescue optimization (SRO) calculation is used for inclusion enhancement. It aims to select the extracted feature set's most relevant and discriminative features, thereby reducing dimensionality and improving subsequent classification efficiency. At long last, bosom disease recognition and characterization are completed utilizing the double-stage spiking convolutional brain network (DSS-CNN). The DSS-CNN model is prepared on the streamlined list of capabilities to characterize mammographic pictures into two classes: harmless and dangerous. Utilizing DL strategies, the model figures out how to recognize unpretentious examples and highlights demonstrative of breast cancer, empowering exact characterization.

### 2.1 ROI Segmentation

The division of regions of interest (ROI) in mammograms is a basic step toward the computerized recognition of bosom malignant growth. For reliable feature extraction and subsequent classification, accurate ROI segmentation is necessary to precisely separate tumor lesions from the breast tissue that surrounds them. In this work, we present the modified dingo optimization (MDO) calculation, a hearty and effective procedure intended to upgrade the exactness of return for capital invested in mammograms. The dingo optimization algorithm (DOA) is propelled by the social way of behaving and hunting systems of dingoes, which are wild canines tracked down in Australia. The original DOA became known for its ease of use and efficiency in resolving challenging optimization issues. However, to tailor it for the specific needs of mammogram image segmentation and we made modifications that improve its convergence speed and accuracy in detecting tumor lesions. To model the social hierarchy of dingo’s, since the antecedents of the search area are unknown, the best agent approach available is target or target hunting. The behavior of dingoes is formulated as follows. Eqs.(**1**-**5**).

**Table:** 

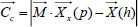	(1)

**Table:** 

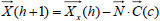	(2)

**Table:** 

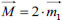	(3)

**Table:** 

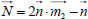	(4)

**Table:** 

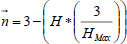	(5)

where addresses the distance between the dingo and prey, addresses the position vector (prey), addresses the position vector (dingo), and addresses the coefficient vector, and addresses the irregular vector in [0,1], addresses the directly decrement from 3 to 0 at each emphasis, addresses the outright worth and duplication with vectors, H addresses the, addresses the Greatest no. of cycle. The dingo can refresh its situation (X, Y) in view of the place of the hunter. For example, 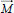 = (1,0) and 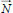 = (1,1), can be achieved through (X^*^ - X,Y^*^) Set up and Dingo. Depending on the status of the best search agent, other dingoes should also update their status. Eqs. (**6**-**11**).

**Table:** 

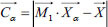	(6)

**Table:** 

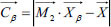	(7)

**Table:** 

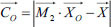	(8)

**Table:** 

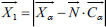	(9)

**Table:** 

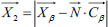	(10)

**Table:** 

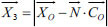	(11)

To compute the concentration of each dingo, subsequent calculations are computed as follows. Eqs. (**12**-**14**).

**Table:** 

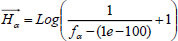	(12)

**Table:** 

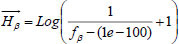	(13)

**Table:** 

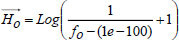	(14)

where addresses the distance between the dingo and prey, addresses the position vector (prey), addresses the position vector of dingo, and addresses the coefficient vector, and addresses the irregular vector in [0,1], addresses the directly decrement from 3 to 0 at each emphasis, addresses the outright worth and duplication with vectors, H addresses the, addresses the greatest number of cycle, addresses the wellness worth of alpha (α) dingo, addresses the wellness worth of alpha (β) dingo, addresses the wellness worth of other dingoes. Assuming there is no announcement, it implies that the dingo has raised a ruckus around town and finished the chase. To formulate the strategy, 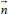 the value decreases linearly. Note that 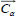 the conversion range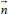 is also reduced. Also 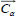 called a random value in the interval [-3n, 3n] where n decreases from 3 to 0 at each iteration. The search agent's next state can be any state between its current state and the victim state when the random values are [1, 1]. In this manner, applied to arbitrary qualities, if the off chance that the worth is not exactly 1, the hunter creates some distance from the looking through specialist, yet assuming the value is more prominent than 1, the pack moves toward the prey. The MDO algorithm builds a solid foundation for precise feature extraction and classification by effectively isolating tumor lesions. This ultimately leads to improved diagnostic outcomes. Algorithm **1** describes the working process of ROI segmentation using MDO.

**Algorithm 1:** ROI segmentation using MDO.

Input: Input images, population size, number of iterations	
Output: ROI target segmentation	
1.	Create initial search agents
2.	Define the initial fitness of each vector 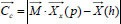
3.	While the Conclusion ailment was not touched do
4.	Appraise each dingo’s fitness and strength cost.
5.	Iteration-1
6	Perform replication
7.	for i= 1: C_in_ do
8.	Renew the modern search mediator status.
7.	End for
8.	Estimating the fitness and strength cost of dingoes
9.	Iteration = Iteration +1
10.	Check if, Iteration 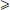 Stopping criteria
11.	Update the final value
12.	End

#### 2.1.1 Importance of MDO Algorithm

Accurate ROI segmentation is important for separating tumor regions from non-tumorous breast tissue. The original Dingo Optimisation Algorithm (DOA), which mimics the cooperative hunting behaviour of Australian dingoes, was applied to medical imaging and enhanced. The MDO algorithm incorporated the following processing stages specifically designed for mammogram segmentation:

Adaptive Exploration and Exploitation Balance: The exploration and exploitation were dynamically balanced in optimization *via* a time-varying control parameter. This leads to a faster convergence process and avoids premature stagnations.

Best-Agent Target Hunting: As the antecedents of the search space are unavailable, the best agent can dynamically lead others foraging towards it to cooperative hunting. All agents are modified using the effect of global best attraction while adding local perturbation to improve search characteristics.

Mutation-Based Perturbation: A low likelihood mutation step was incorporated in order to move out of local minima, particularly for dense tissue areas and low contrast mammograms.

Imaging-Aware Fitness Function: A new composite objective function using the gradient magnitude, region homogeneity, and compactness is defined to measure candidate segmentations. This guarantees well-defined edges of the tumor and avoids over-segmentation.

Multi-Scale Initialization: Agents initialized with multi-scale pre-processed maps decreased the sensitivity of results to the initial position, in addition to leading to faster convergences.

These changes enabled MDO to obtain accurate and stable segmentation on tumor boundaries even for low-contrast mammograms.

#### 2.1.2 Findings of the MDO Algorithm

Improved Segmentation Accuracy: The proposed MDO algorithm significantly improves the accuracy of the ROI segmentation in mammograms by automatically choosing an optimal threshold and contour parameters for proper tumor boundary detection.

Enhanced Exploration–Exploitation Balance: Through adaptive control parameters and a nonlinear convergence factor, the MDO approach achieves a better trade-off between exploration (global search for potential boundaries) and exploitation (local enhancement), leading to more fast yet stable convergence than that exists in basic DOA.

Resistance to Local Minima: The new scheme with dynamically changing step and search direction can avoid the premature convergence, allowing the algorithm to escape local minima and locate the more accurate tumor region that exists in complex mammographic texture.

High Robustness and Stability: Experimental results show that MDO can achieve stable performance under different image scales and noise levels, which indicates that it is robust and reliable for 3D medical image analysis.

Reduced Computational Complexity: The simplified fitness update mechanism and selective parameter tuning in MDO reduce computation costs while maintaining high segmentation accuracy, which is favourable for real-time or large-scale medical image analysis.

Superior Performance over Conventional Methods: The comparative analysis shows that MDO has superior performance over classical segmentation techniques, including Otsu thresholding, K-means clustering algorithm, and conventional metaheuristic algorithms like PSO, GWO, and GA for Dice coefficient, Jaccard index and sensitivity.

Effective Feature Preservation: The algorithm is designed to keep distinctive edge and texture features of tumor regions, which are essential in preserving the detailed structures of tumor cells, therefore advantageously contributing to improved downstream feature extraction and classification performance in our proposed detection framework.

### 2.2 Feature Extraction

Feature extraction is the process of converting raw data into a collection of useful features that may be efficiently applied to additional analysis, like pattern recognition or classification. In the context of mammogram analysis, feature extraction involves identifying and quantifying important characteristics of the images, such as shapes, textures, and edges, that are indicative of potential tumor regions. U-Net is a convolutional neural network architecture originally designed for biomedical image segmentation. It is particularly effective for feature extraction in medical images due to its ability to capture fine details and context at different scales. The architecture consists of an encoder and decoder, which together allow the network to learn rich and hierarchical features from the input images.

- Normalize the mammogram images to ensure consistent input data. Resize the input images to match the input size expected by the U-Net model.
- Apply a series of convolutional layers with ReLU activation to extract low-level features. Use max pooling layers to downsample the image, reducing the spatial dimensions while increasing the depth. This helps in capturing context at different scales. At each stage, the network learns increasingly abstract features, capturing edges, textures, and shapes.
- At the bottom of the U-Net, a series of convolutional layers with a larger number of filters is applied to capture dense and complex features.
- Use transposed convolution layers to increase the spatial dimensions of the feature maps, making them suitable for segmentation tasks. Integrate skip connections from the layers in the encoder path. These connections help in combining high-resolution features from the encoder with the up-sampled features, retaining fine details.
- The features from the skip connections and the up-sampling layers are fused to create detailed feature maps. Take the feature maps out of the decoder path's intermediary layers. Rich details about various aspects of the input image are included in these maps.
- The output feature maps from the final layer of the decoder are used as the deep features for subsequent analysis. These features encapsulate both the spatial and contextual features from the mammograms.
- The search and rescue optimization (SRO) algorithm is used to reduce the dimensionality of the feature vectors while retaining the most important information.

### 2.3 Feature Optimization

Feature optimization is the method involved with choosing the most pertinent and instructive elements from a dataset to work on the presentation of an AI model. The goal is to reduce the dimensionality of the data while retaining the features that contribute most to the prediction possibility of the model. This aids in working on computational effectiveness, decreasing overfitting, and upgrading the model's accuracy and interpretability. Search and rescue optimization (SRO) is a metaheuristic calculation propelled by this present reality search and rescue tasks, where a group of heroes looks for survivors in a fiasco-stricken region. It imitates the techniques utilized in these activities, like investigation, abuse, and data division between colleagues, to proficiently track down the ideal arrangement. The value of the signals found at that location indicates the suitability of the optimization problem's solution, which corresponds to the population's location. In the SRO calculation, the places of the left person are put away in the memory lattice (grid A), and the human positions are put away in the position network (framework B). Array A and Array P share the same dimensions. These are B×C groups, where C is the size of the issue, and B is the number of colleagues. The pursuit grid (lattice T) is the framework containing the places of the distinguished hints. This group comprises two groups, X and M. All new arrangements at the social and individual levels are based on SAR's Center Piece of information Lattice. These lattices (B, A, D) are refreshed at each step of the human hunt.

**Table:** 

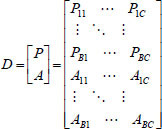	(15)

where An and P are memory and perceptual state grids, separately, and B is the main layered character state Eq. (**15**). The social and individual phases of human search, respectively, are depicted. As indicated by the past clarification, it is utilized to get an inquiry heading by thinking about irregular references among the discovered references.

**Table:** 

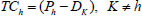	(16)

Where *P*_*h*_ , *D*_*k*_ and *TC*_*h*_ K is a random integer between 1 and 2B. Since h=K, *D*_*k*_ is the same *P*_*h*_, K is chosen by K ≠ h Eq. (**16**). Team members generally try to avoid multiple location searches. As a result, the search ought to be carried out in such a way that the gang members' movements are controlled by one another. A binomial hybrid administrator was utilized to take advantage of this impediment. As made sense of in the past segment, on the off chance that the apparent sign is more important than the signal associated with the current state. The new state of the H-Man comes in all dimensions.

**Table:** 

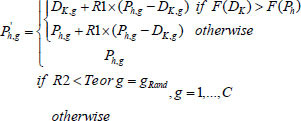	(17)

where 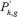 is the new level of the gth dimension for the hth man Eq. (**17**). *D*_*kg*_ , Cth dimension position for Kth clue. *F*(*D*_*K*_) And *F*(*P*_*h*_) R1 is a random number with uniform distribution in the range [-1, 1]. R2 is a random number uniformly distributed in the range [0, 1]. R2 is different for each measurement, but R1 is constant for all measurements *g*_*Rand*_. is a random integer between 1 and C, 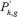 ensures that at least one dimension is different 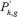. Te is an algorithm parameter between 0 and 1. In the individual phase, people search for their current situation, and in this phase, the concept of connecting different cues is used, which is used in the social phase. Got a new position as H-Man.

**Table:** 

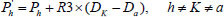	(18)

Where K and a are random integers that fall between 1 and 2B Eq. (**18**). These numbers are chosen so that h K a doesn't get confused with other signs. R3 is an irregular number with a uniform dispersion somewhere in the range of 0 and 1. Choices about friendly and individual activities should be made and changed, assuming that they exist.

**Table:** 

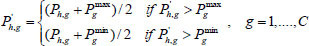	(19)

It is used to change the new position of h-man. where *P*_*g*_^*max*^ and *P*_*g*_^*min*^ are the jth dimension's maximum and minimum limits, respectively (Eq. **19**). In every cycle, the gathering individuals are looked at by these two stages, and after each step, on the off chance that the goal capability esteem at position 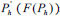 is greater than the previous position (*F*(*P*_*h*_)), the previous position (*P*_*h*_) is randomly stored. The memory matrix level (A) is used. Eq. (**20**).

**Table:** 

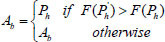	(20)

Position will be acknowledged as a new location using Eq. (**21**).

**Table:** 

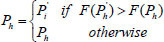	(21)

Where *A*_*b*_ is the area of the b-th put-away pointer in the memory lattice. b is an irregular number between 1 and B. The bombed search number (USN) is initially set to 0 for each colleague. Assuming an individual finds a critical follow in the first or second period of the pursuit, that individual's USN is set to 0; otherwise, 1 point will be added as displayed

**Table:** 

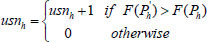	(22)

This *usn*_*h*_ suggests that H-Man has yet to find any important clues Eq. (**22**). If an individual's USN exceeds MU, it moves to one more situation in the pursuit space. For obliged improvement issues, if USN > MU, the arrangement in the hunt space meets in an irregular answer for a possible arrangement. Eq. (**23**)

**Table:** 

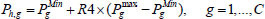	(23)

Also, for an incomplete solution, if USN > MU, if an answer that disregards the base number imperative is chosen in the memory grid, the ongoing arrangement is changed over completely to that arrangement, and the ongoing arrangement is put in the memory framework. R4 is an irregular number with a uniform conveyance somewhere in the range of 0 and 1. As indicated by this technique, for a boost issue, one arrangement is better compared to another if the accompanying circumstances are met: (Eq. **24**)

**Table:** 

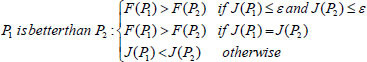	(24)

Where ε is the adjustment vector to optimize the size of *via*ble space.

**Table:** 

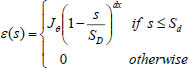	(25)

Where s is the parameter ε and dx is a parameter controlling the rate of space reduction. (Eq. **25**)

**Table:** 

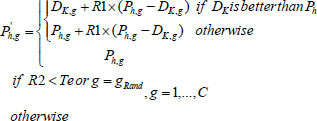	(26)

**Table:** 

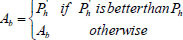	(27)

**Table:** 

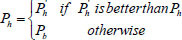	(28)

**Table:** 

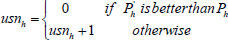	(29)

where *D*_*k*_ or *P*_*h*_, are better than *P*_*h*_ if and only content. Algorithm **2** gives the process of working feature optimization using SRO. Eqs. (**26**-**29**).

**Algorithm 2:** Feature optimization using SRO.

Input: Number of features, maximum iteration, and threshold condition	
Output: Best optimal features	
1.	Randomly initialize a population
2.	Compute current best position (Xbest)
3.	Define search direction using random references 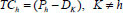
4.	While the stop standard is not satisfied, do
5.	For i=1 to N do
6	Compute social and personal actions 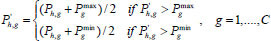
7.	The memory matrix level (A) is used. 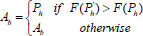
8.	The person's USN is, 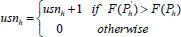
9.	Update *usn*_*h*_
10.	Compute the optimal solution to search space converges 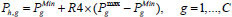
11.	The ε limitation gearshifts the size of the *via*ble space. 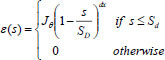
12.	Define the ε-constrained fitness function 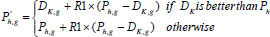
13.	Update the fitness values
14.	End if
15.	Apply the restart strategy
16.	Find the present best position and update Xbest
17.	End while
18.	Return Xbest

#### 2.3.1 Modification of SRO for the Proposed Work

The standard SRO was adapted and modified to optimize the features by a deep feature, which is extracted from the U-Net segmentation network. The conventional SRO, which was initially developed for continuous optimization, was reformulated as a binary–real hybrid model to facilitate the feature selection and feature weighting problems simultaneously. Binary masks formed the position vector of each rescuer to encode selected features, and an adaptive thresholding function was also proposed to map the continuous position values into binary action choices. Modified the fitness to simultaneously optimize the two objectives of maximizing an aggregated classification accuracy obtainable by the VGG-19 classifier and minimizing the number of selected features, effectively trading performance with feature selection. Furthermore, an ε-constrained adaptive approach was improved to regulate the tradeoff between exploration and exploitation according to population cosine diversity to avoid premature convergence. A restarting scheme was applied to reset stagnating agents with a view to maintaining the global search ability in high-dimensional feature space. These adaptations allowed SRO to effectively locate the most discriminative U-Net features, with better classification performance, computational efficiency as well and model interpretability for breast cancer detection.

#### 2.3.2 Important Findings of SRO

Efficient Feature Selection: SRO is capable of effectively discovering the most discriminative and instructive features from the high-dimensional feature space computed by U-Net, while remarkably alleviating redundancy and computation cost without compromising diagnostic accuracy.

Balanced Exploration and Exploitation: The cooperative search in SRO precisely means to find a balance between exploration (global search for new candidate feature subsets) and exploitation (further refinement of the promising subspaces), which promotes convergence to the feature subset with maximum discrimination strength.

Superior Convergence Characteristics: Compared with conventional swarm-based optimizers such as PSO, GWO, and WOA, SRO generally shows a faster rate of convergence and is more stable in finding the optimal solutions due to its adaptive ε-constrained strategy and efficient memory matrix update technique.

Enhanced Classification Accuracy: The feature subset selected by SRO maximizes the classification capability of the VGG-19 model, which can further increase the signal-to-noise ratio of input features and thus provides higher sensitivity and specificity for tumour classification.

Avoidance of Local Minima: By multi-agent cooperation and a restart mechanism, SRO can reduce the possibility of premature convergence and let the algorithm delve into enough solution space to find a more accurate optimization for every feature.

Improved Generalization and Interpretability: Through removing irrelevant or noisy features, SRO not only enhances the model generalizability on unseen mammogram datasets but also emphasizes on most biologically meaningful feature regions for better interpretability.

Scalability Across Modalities: The generality of SRO enables it to be seamlessly incorporated into various deep learning models and imaging modalities (mammography, MRI, CT, and histopathology), confirming the versatility of our model in heterogeneous domain cancer detection tasks.

### 2.4 Breast Tumour Detection and Classification

Breast tumor detection and classification involve identifying abnormal tissue regions in mammographic images and determining whether the detected tumor is benign or malignant. Accurate and early detection plays a vital role in enabling timely diagnosis and effective treatment planning. The application of advanced deep learning (DL) techniques, such as the Dual-Stage Spiking Convolutional Neural Network (DSS-CNN), can substantially improve both detection sensitivity and classification accuracy by effectively capturing spatial and temporal features from breast images. The DSS-CNN is a hybrid neural network architecture that combines the principles of a spiking neural network (SNN) and a convolutional neural network (CNN). SNNs mimic the way neurons in the human brain communicate through discrete spikes, which makes them energy-efficient and suitable for processing temporal data. CNNs are highly effective in image processing tasks due to their ability to learn spatial hierarchies of features through convolutional layers. Initially, a solo DSS-CNN was utilized to extract boost highlights for each information picture. Direct backslide models were created to predict DSS-CNN properties. The transformation layer that is utilized to simulate the visual cortex's information integration system is located in the second layer of DSS-CNN. Eqs. (**30**, **31**).

**Table:** 

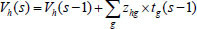	(30)

**Table:** 

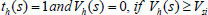	(31)

Only the neuron with the underlying spike duration is permitted to fire at each position of the component maps due to a hindrance system in the convolutional layer, is the synaptic weight between the h-th neuron's spikes and the g-th input in the neuron's receptive field. represents the voltage of the h-th IF neuron at phase s:

**Table:** 

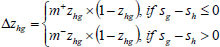	(32)

where signifies the adjustment of weight, the information rates (set at 0.004 and 0.003, respectively)23, and the spike seasons of the h neuron and the g neuron's feedback spikes, individually (Eq. **32**). The learning crossing point is imparted as follows. (Eq. **33**)

**Table:** 

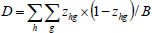	(33)

where the complete number of synaptic loads is addressed by B. The convolutional layer stops when C falls under 0.01. Following setting up the DSS-CNN, as far as possible V-th is set to perpetuation, and the last step voltage estimate in every neuron is figured as DSS-CNN normal for the visual improvement. Since voltages in convolutional neurons aggregate over the long haul and never recover when V-th becomes limitless, the last voltage values mirror the action of the DSS-CNN. The goal capability of the relapse model is characterized as follows: (Eqs. **34**,**35**)

**Table:** 

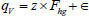	(34)

**Table:** 

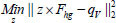	(35)

where is the fMRI reaction of voxel V, z is the relapse model's weight boundaries, and the element vector at the location (h, g) of the SCNN feature maps is represented. The likelihood R of the noticed fMRI reaction is given as the pre-picture t, which can be addressed as a multivariate Gaussian distribution.

**Table:** 

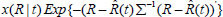	(36)

**Table:** 

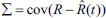	(37)

where 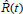 indicates the anticipated fMRI reaction t, and Σ means the clamor covariance network for the train tests Eqs. (**36**,**37**). The following function is then used to determine the noise's standard deviation. (Eq. **38**)

**Table:** 

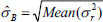	(38)

where 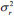
indicates the changeability of reactions across 35 sequential meetings for each test picture. Then, at that point, we determined the reaction fluctuation by deducting the commotion change from the mean reaction difference.

**Table:** 

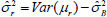	(39)

where 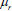 responses to each test over consecutive sessions are indicated here (Eq. **39**). Algorithm **3** describes the working process of breast cancer detection and classification using DSS-CNN.

**Algorithm 3:** Breast cancer detection and classification using DSS-CNN.

Input: Population size, Benign threshold, Malignant threshold	
Output: Classes – Benign and Malignant	
1.	Begin;
2.	Initialize the step sizes of the population
3.	Define the field and emits a spike 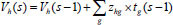
4.	Compute learning junction, 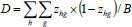
5.	Found the objective function of the regression model 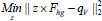
6	End if
7.	Else
8.	Compute the standard deviation of the noise 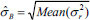
7.	Find the best fitness value
8.	End if
9.	Compute the response variance by subtracting the noise variance from the mean 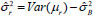
10.	Else
11.	End While
12.	Find the best output value

### 2.5 Experimental Setup Requirements

Used PyCharm outline 2020 from adaptation 1.4.0 and Python 3.6, calling CUDA, Cudnn, OpenCV, and other important libraries. The test setup includes a GeForce GTX 1080Ti 11GB graphics card and Linux Ubuntu 16.04. The exhibition of proposed and existing works is dissected through various measurements, for example, exactness, accuracy, review, F-measure, misleading positive rate (FPR), bogus negative rate (FNR), Matthews relationship coefficient (MCC), and normal mean accuracy (Guide).

### 2.6 Description of Dataset

**Fig. (2a,b):**
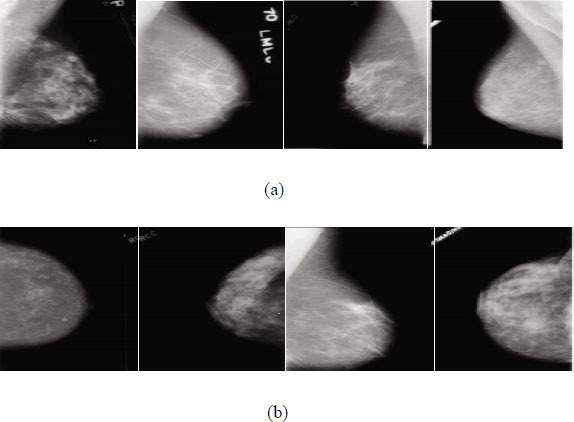
Test samples of the CBIS-DDSM dataset.

The Curated Breast Imaging subset of DDSM (CBIS-DDSM) dataset includes 10239 pictures, including whole mammograms, altered, and profit from introductory capital venture cover pictures with mass and calcification are used. 2424 complete mammograms of harmless and dangerous masses were utilized in this review. Information expansion is a procedure utilized in picture handling to create additional preparation information from current information. Expansion is finished in this concentrate by flipping pictures on a level plane, pivoting them at 90 and 180 degrees, and making different duplicates. After the increase, a sum of 4865 pictures were delivered. 60% of the data is used for planning, 30% is used for endorsement, and 10% is used for testing. Because of the plans taking care of the unit, we chose to create our model, which can't manage huge pictures, and the size of the photos has been decreased from 3000×4500 to 1000×2000 pixels. Table **2** describes the summary of the CBIS-DDSM dataset. It contains a total of 4,865 mammography images, with 2,545 benign cases and 2,320 malignant cases. The dataset is partitioned into preparation, approval, and testing sets. The preparation set comprises 2,919 pictures, with 1,425 harmless cases and 1,494 dangerous cases. The approval set contains 1,459 pictures, with 895 harmless and 564 threatening cases. This set is utilized to assess the presentation of the prepared models during the preparation cycle and to adjust the hyperparameters. The testing set includes 487 images, with 225 benign cases and 262 malignant cases. The CBIS-DDSM subset was selected and curated by trained mammographers to provide a well-annotated and accessible dataset for the training and testing systems in mammography (Fig. **2**).

## 3 RESULTS AND DISCUSSION

A detailed evaluation of the proposed methodology is provided in the Results and Discussion section as follows. These findings illustrate how the model improves tumor detection and classification while addressing critical challenges observed in current diagnostic techniques.

### 3.1 Result Analysis of Segmentation Algorithms

**Fig. (4):**
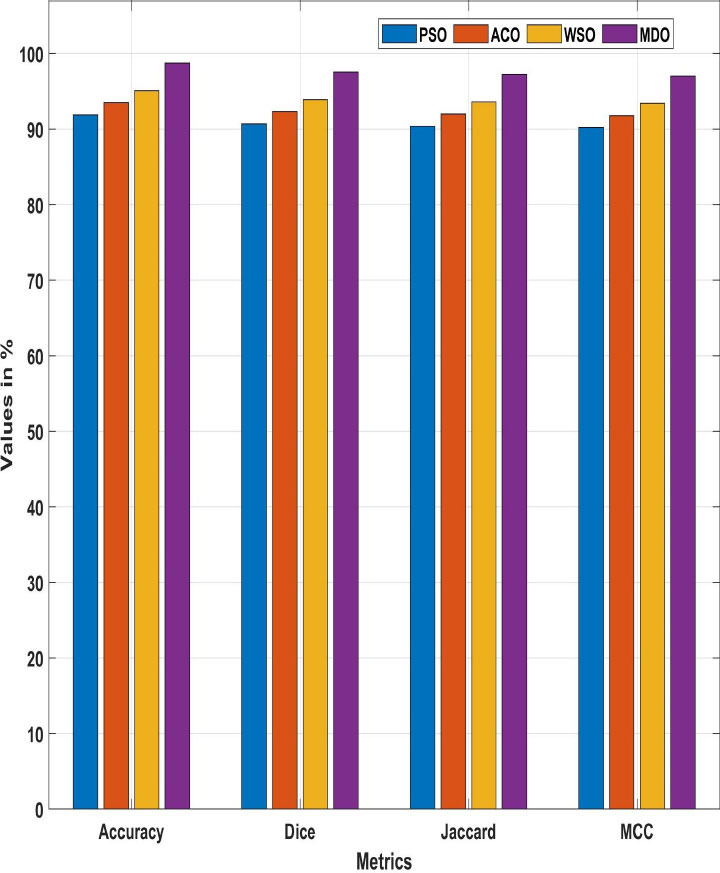
Results comparison of different segmentation algorithms.

In Fig. (**3**) the performance of various segmentation algorithms is shown and evaluated using several metrics, including accuracy, Dice coefficient, Jaccard index, and Matthews correlation coefficient (MCC). Table **3** describes the results indicate significant differences in performance across the algorithms, with the modified dingo optimization (MDO) algorithm showing superior results. Starting with accuracy, the MDO algorithm achieved 98.758%, which is 2.652% higher than the whale swarm optimization (WSO) algorithm at 95.106%, 5.254% higher than the ant colony optimization (ACO) algorithm at 93.504%, and 6.856% higher than the particle swarm optimization (PSO) algorithm at 91.902%. The substantial improvement shows the effectiveness of MDO in accurately segmenting the regions of interest. In terms of the dice coefficient, the MDO algorithm again outperformed the others, achieving 97.568%. This represents a 3.652% increase over WSO's 93.916%, a 5.254% increase over ACO's 92.314%, and a 6.856% increase over PSO's 90.712%. The Dice coefficient improvement shows MDO's precision in overlapping regions between the predicted and actual segmentation. When examining the Jaccard index, MDO achieved 97.259%, which is 3.652% higher than WSO's 93.607%, 5.254% higher than ACO's 92.005%, and 6.856% higher than PSO's 90.403%. The Jaccard index results confirm that MDO has a higher agreement between the segmented regions and the ground truth. Finally, the MCC values show that MDO reached 97.056%, which is 3.652% higher than WSO's 93.404%, 5.254% higher than ACO's 91.802%, and 6.856% higher than PSO's 90.200%. The MCC improvement indicates that MDO performs better in binary classification tasks within the segmentation process. Figure **4** highlights the robustness of MDO in segmenting regions of interest in mammograms, making it a superior choice for this task.

### 3.2 Result Analysis of Proposed and Baseline Models

**Fig. (5):**
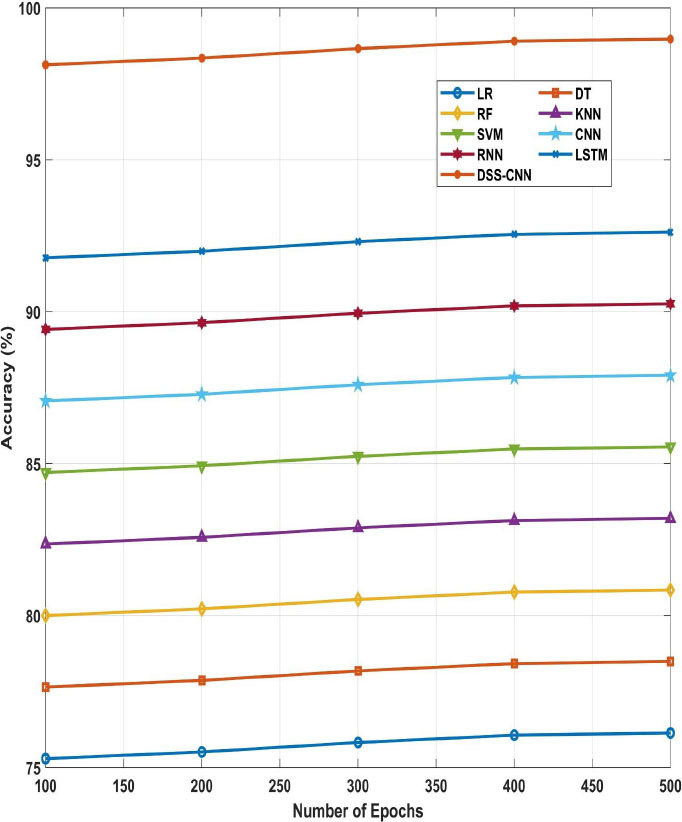
Accuracy comparison with the number of epochs.

Table **4** describes the comparative analysis of proposed and baseline models for breast cancer detection and classification for the CBIS-DDSM dataset with varying epochs. The results of the proposed DSS-CNN model are compared with the baseline models, such as logistic regression (LR), decision tree (DT), random forest (RF), K-nearest neighbor (KNN), support vector machine (SVM), convolutional neural network (CNN), recurrent neural network (RNN), and long short-term memory (LSTM). Figure **5** shows the accuracy improvements of each model with an increasing number of epochs, highlighting the learning and convergence capabilities of the models. Starting with an accuracy of 100 epochs, LR showed a gradual increase, reaching 76.136% at 500 epochs. This is an overall improvement of 0.843%, indicating that while LR benefits from more epochs, the gains are relatively modest. The DT model's accuracy increased from 77.647% at 100 epochs to 78.490% at 500 epochs, showing a total improvement of 0.843%. Similar to LR, the DT model's improvement is steady but limited, suggesting early saturation in learning. The RF model exhibited an accuracy increase from 80.001% at 100 epochs to 80.844% at 500 epochs, a total gain of 0.843%. RF shows slightly better performance improvement compared to LR and DT, though the overall increase remains modest. The KNN model shows a more pronounced improvement, increasing from 82.355% at 100 epochs to 83.198% at 500 epochs, a total gain of 0.843%. This suggests better adaptability with more epochs but still within a limited range. Starting at 84.709% accuracy with 100 epochs, SVM's accuracy enhances to 85.552% at 500 epochs, with a total increase of 0.843%. The performance of SVM reflects an improvement similar to KNN. The CNN model's accuracy improved from 87.063% at 100 epochs to 87.906% at 500 epochs, showing a total increase of 0.843%. CNNs benefit significantly from more epochs due to their capacity to learn complex patterns. RNN model started at 89.417% accuracy at 100 epochs and improved to 90.26% at 500 epochs, with a total gain of 0.843%. RNNs show benefits from additional epochs, reflecting their ability to capture temporal dependencies effectively. LSTM showed a marked improvement from 91.771% at 100 epochs to 92.614% at 500 epochs, with a total increase of 0.843%. It highlights LSTM's superior learning capability over time. The DSS-CNN model exhibited a significant improvement, starting at 98.125% accuracy at 100 epochs and reaching 98.968% at 500 epochs, with a total gain of 0.843%. It indicates that DSS-CNN uses the extended training to achieve exceptional accuracy, outperforming all other models.

**Fig. (6):**
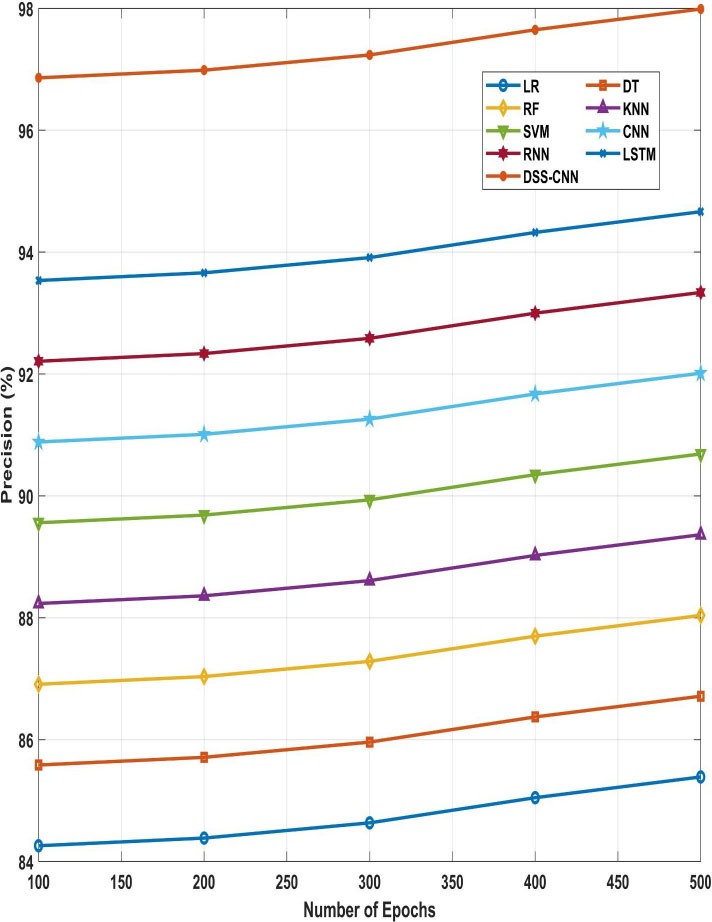
Precision comparison with the number of epochs.

Figure **6** highlights the precision improvements of each model as the number of epochs increases, showing their learning efficiency and ability to reduce false positives. Starting at precision with 100 epochs, LR's precision increased to 85.388% at 500 epochs, reflecting an improvement of 1.129%. This steady improvement shows LR's capacity to enhance precision with more training, albeit at a moderate rate. The DT model's precision increased from 85.584% at 100 epochs to 86.713% at 500 epochs, a total gain of 1.129%. Similar to LR, DT shows a steady but modest improvement in precision with additional training epochs. The RF model exhibited precision improvement from 86.909% at 100 epochs to 88.038% at 500 epochs, a total increase of 1.129%. RF shows better precision gains compared to LR and DT, benefiting more significantly from extended training. KNN's precision increased from 88.234% at 100 epochs to 89.363% at 500 epochs, showing a total improvement of 1.129%. This indicates that KNN effectively enhances precision with more training epochs. SVM's precision rose from 89.559% at 100 epochs to 90.688% at 500 epochs, with a total increase of 1.129%. SVM shows a consistent and noticeable improvement in precision with additional epochs. The CNN model's precision improved from 90.884% at 100 epochs to 92.013% at 500 epochs, a total gain of 1.129%. CNNs benefit significantly from more training epochs, reflecting their capacity to learn complex patterns. RNN's precision started at 92.209% at 100 epochs and increased to 93.338% at 500 epochs, a total improvement of 1.129%. RNNs show benefits from extended training, effectively capturing temporal dependencies to enhance precision. LSTM showed marked improvement from 93.534% at 100 epochs to 94.663% at 500 epochs, with a total increase of 1.129%. This underscores LSTM's superior learning capability over time and its effectiveness in enhancing precision. DSS-CNN exhibited the most significant improvement, starting at 96.859% precision at 100 epochs and reaching 97.988% at 500 epochs, a total gain of 1.129%. DSS-CNN uses extended training to achieve exceptional precision, outperforming all other models.

**Fig. (7):**
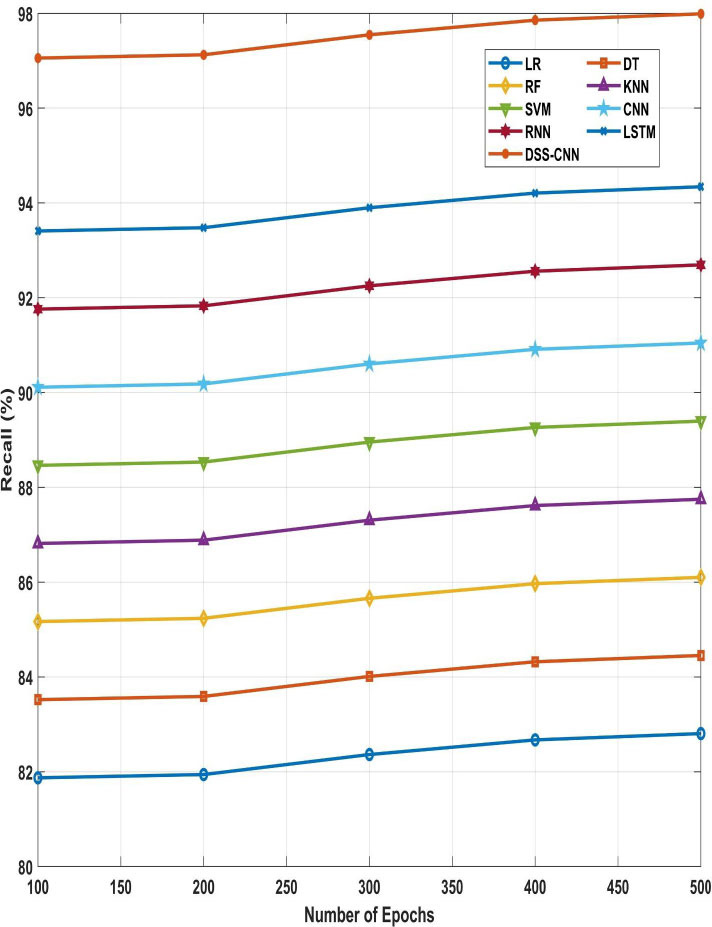
Recall comparison with the number of epochs.

Figure **7** highlights the recall improvements of each model as the number of epochs increases, showing their ability to correctly identify relevant instances. Starting at 81.872% recall with 100 epochs, LR's recall increased to 82.805% at 500 epochs, reflecting an overall improvement of 0.933%. This modest increase indicates that LR improves recall with more training, but the gains are relatively small. The DT model's recall increased from 83.520% at 100 epochs to 84.453% at 500 epochs, a total gain of 0.933%. Similar to LR, DT shows a steady but moderate improvement in recall with additional training epochs. The RF model exhibited recall improvement from 85.168% at 100 epochs to 86.101% at 500 epochs, a total increase of 0.933%. RF shows better recall gains compared to LR and DT, benefiting more from extended training. KNN's recall increased from 86.816% at 100 epochs to 87.749% at 500 epochs, showing a total improvement of 0.933%. This indicates that KNN effectively enhances recall with more training epochs. SVM's recall rose from 88.464% at 100 epochs to 89.397% at 500 epochs, with a total increase of 0.933%. SVM shows consistent and noticeable improvement in recall with additional epochs. The CNN model's recall improved from 90.112% at 100 epochs to 91.045% at 500 epochs, a total gain of 0.933%. CNNs benefit significantly from more training epochs, reflecting their capacity to learn complex patterns. RNN's recall started at 91.760% at 100 epochs and increased to 92.693% at 500 epochs, a total improvement of 0.933%. RNNs show substantial benefits from extended training, effectively capturing temporal dependencies to enhance recall. LSTM showed marked improvement from 93.408% at 100 epochs to 94.341% at 500 epochs, with a total increase of 0.933%. This underscores LSTM's superior learning capability over time and its effectiveness in enhancing recall. DSS-CNN exhibited a significant improvement, starting at 97.056% recall at 100 epochs and reaching 97.989% at 500 epochs, a total gain of 0.933%. DSS-CNN leverages extended training to achieve exceptional recall, outperforming all other models.

**Fig. (8):**
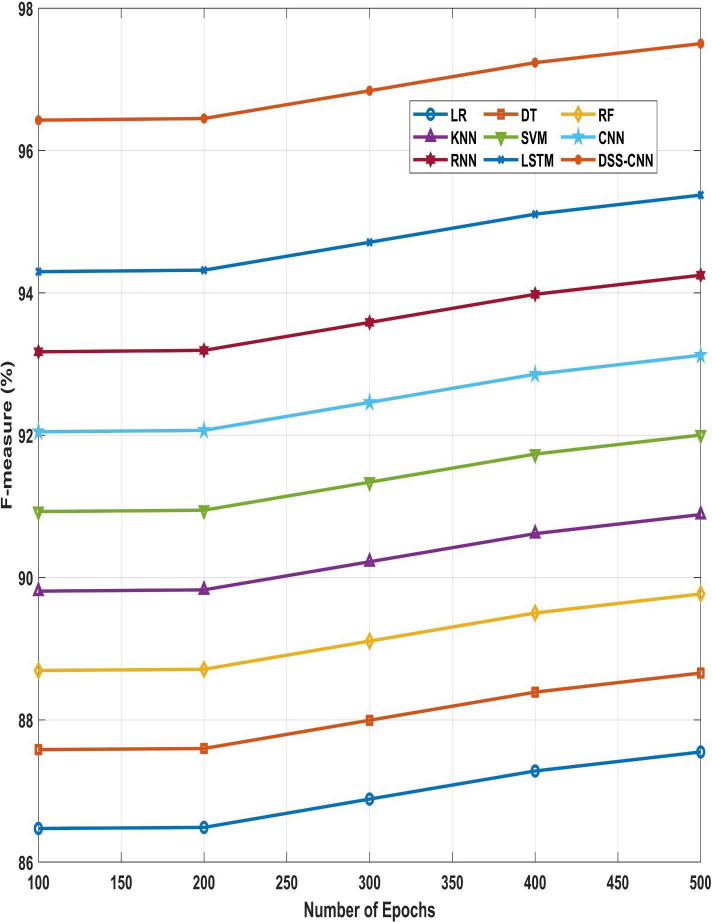
F-measure comparison with the number of epochs.

Figure **8** shows the improvements in F-measure for each model with an increasing number of epochs, reflecting their overall performance in classification tasks. Starting at an F-measure of 86.473% with 100 epochs, LR's F-measure increased to 87.548% at 500 epochs, reflecting an overall improvement of 1.075%. This gradual increase indicates that LR benefits from more training but shows relatively modest gains. The DT model's F-measure increased from 87.581% at 100 epochs to 88.657% at 500 epochs, a total gain of 1.076%. DT shows a steady improvement in F-measure with additional training epochs, similar to LR. The RF model exhibited an increase in F-measure from 88.693% at 100 epochs to 89.769% at 500 epochs, with a total gain of 1.076%. RF demonstrates better performance improvement compared to LR and DT, indicating its robustness with more training. KNN's F-measure improved from 89.808% at 100 epochs to 90.884% at 500 epochs, showing a total increase of 1.076%. It reflects KNN's effective enhancement in F-measure with extended training epochs. SVM's F-measure rose from 90.926% at 100 epochs to 92.002% at 500 epochs, with a total improvement of 1.076%. SVM shows a consistent and noticeable increase in F-measure with additional epochs, highlighting its capacity to learn complex patterns. The CNN model's F-measure improved from 92.047% at 100 epochs to 93.123% at 500 epochs, a total gain of 1.076%. CNNs benefit significantly from more training epochs, reflecting their ability to capture intricate details in data. RNN's F-measure started at 93.171% at 100 epochs and increased to 94.247% at 500 epochs, a total improvement of 1.076%. RNNs show substantial benefits from extended training, effectively capturing temporal dependencies to enhance performance. LSTM shows marked improvement from 94.297% at 100 epochs to 95.373% at 500 epochs, with a total increase of 1.076%. This underscores LSTM's superior learning capability over time and its effectiveness in improving the F-measure. DSS-CNN exhibited the most significant improvement, starting at 96.426% F-measure at 100 epochs and reaching 97.501% at 500 epochs, a total gain of 1.075%. DSS-CNN leverages extended training to achieve an exceptional F-measure, outperforming all other models.

### 3.3 Result Analysis of Proposed and Existing State-of-the-art Models

The performance of various models for breast cancer detection and classification was evaluated using the CBIS-DDSM dataset. Table **5** provides a detailed comparative analysis of these models based on several metrics. The accuracy of the proposed DSS-CNN model is 98.598%, which is significantly higher than the existing models. For instance, DSS-CNN surpasses M-YOLOv5x, the next best model, by 2.095%. Compared to O-YOLOv5s, DSS-CNN shows an 8.097% increase in accuracy, shows its superior performance in accurately classifying breast cancer cases. DSS-CNN achieves a precision of 97.343%, which is 0.341% higher than M-YOLOv5x's 97.002%. The improvement is more pronounced when compared to O-YOLOv5s, with DSS-CNN exhibiting a 6.441% increase. This indicates DSS-CNN's effectiveness in minimizing false positives. The sensitivity of DSS-CNN is 97.515%, which outperforms M-YOLOv5x by 1.468%. When compared to the least sensitive model, O-YOLOv5m, which has a sensitivity of 91.454%, DSS-CNN shows a significant 6.061% increase. This improvement highlights DSS-CNN's ability to correctly identify true positive cases of breast cancer.

**Fig. (9):**
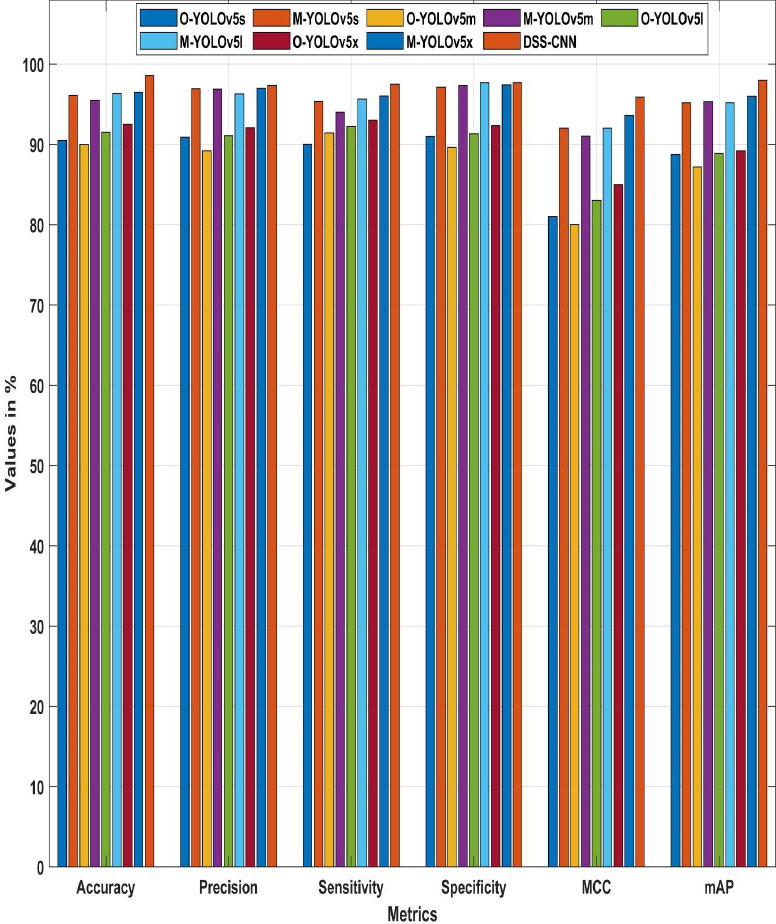
Results comparison of proposed and existing state-of-the-art models based on accuracy, precision, sensitivity, specificity, MCC, and mAP.

**Fig. (10):**
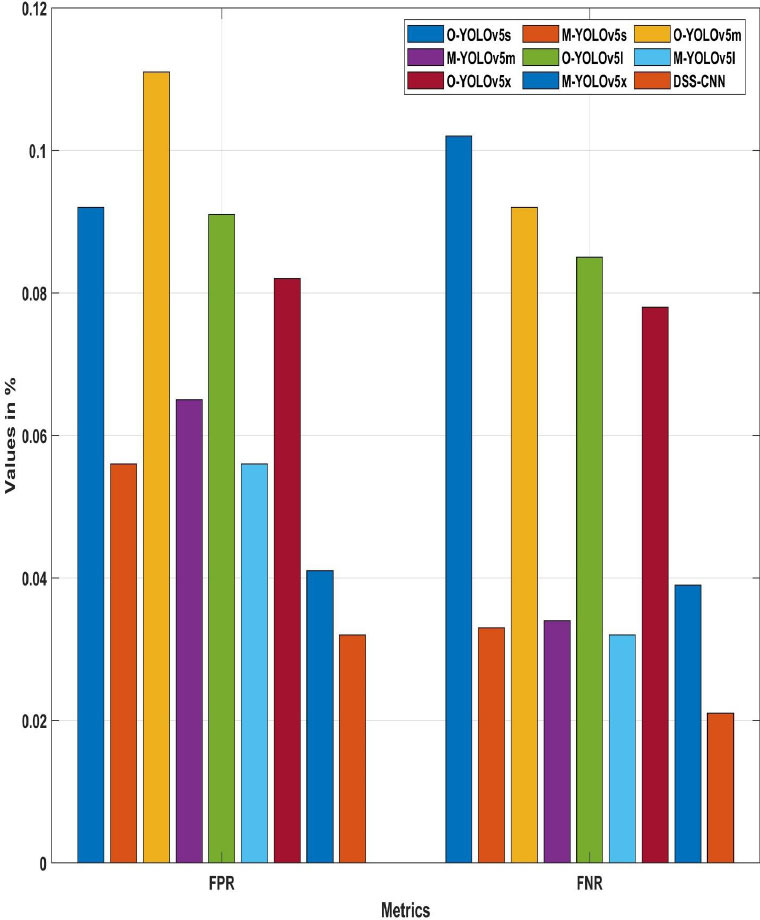
Results comparison of proposed and existing state-of-the-art models based on FPR and FNR.

DSS-CNN achieves a specificity of 97.689%, which is 0.260% higher than M-YOLOv5x’s 97.429%. Compared to the specificity of O-YOLOv5s at 91.023%, DSS-CNN shows a 6.666% improvement. This indicates DSS-CNN's efficacy in correctly identifying non-cancerous cases. The MCC for DSS-CNN is 95.898%, which surpasses M-YOLOv5x by 2.289%. The increase is even more significant when compared to O-YOLOv5m, with DSS-CNN showing a 15.873% higher MCC. This metric emphasizes DSS-CNN's balanced accuracy in both positive and negative classifications. DSS-CNN has a mAP of 97.989%, which is 1.982% higher than M-YOLOv5x’s 96.007%. When compared to O-YOLOv5m's 87.209%, DSS-CNN achieves a 10.780% increase, indicating its superior overall detection performance. DSS-CNN significantly reduces the FPR to 0.032, which is 0.009 lower than M-YOLOv5x's 0.041. Compared to the highest FPR of 0.111 in O-YOLOv5m, DSS-CNN shows a reduction of 0.079, highlighting its ability to minimize false alarms. DSS-CNN achieves the lowest FNR of 0.021, which is 0.011 lower than M-YOLOv5x's 0.032. The improvement is pronounced compared to O-YOLOv5m, with DSS-CNN shows a reduction of 0.071. It underscores DSS-CNN's effectiveness in reducing missed detections of breast cancer cases. Figures **9** and **10** show that the proposed DSS-CNN model performs superior performance across all evaluated metrics compared to existing state-of-the-art models.

### 3.4 Implications for Future Research & Clinical Practice

The proposed MDO-U-Net-SRO-DSS-CNN framework represents a step towards intelligent, data-driven breast cancer detection systems. The results presented illustrate the promise of optimization-based learning with deep architectures to produce high diagnostic accuracy, adaptability, and computational efficiency. Future work: The proposed integrated framework provides opportunities for developing adaptive and explainable AI models trained on multimodal imaging data, such as mammography, ultrasound, or MRI, that are expected to be robust across clinical datasets. And, if deployed with an FPGA, it provides an on-site screening/diagnosis tool capable of being included in fast-track clinical decision-making thanks to a minimum latency. Clinically, AI-based systems could act as 'smart' diagnostic tools by giving immediate and reliable assessments; they can also assist radiologists in early diagnosis, risk screening, and treatment planning. With accuracy, interpretability, and real-time usability warranted, this hybrid optimization–deep learning scheme holds great promise in furthering new frontiers in precision oncology and spearheading future generation computer-aided diagnostic (CAD) systems for breast cancer screening and prognosis.

### 3.5 AI Systems and Clinical Decision Support in Real Time

Finally, the MDO-based U-Net, SRO, and DSS-CNN fusion methods pave the way for real-time AI systems where medical data could be analyzed in situ during clinical examinations. This allows for real-time diagnostic information, helping radiologists make quicker and more accurate decisions at the point of care. Using hardware acceleration *via* FPGA, or GPU-based computing, the system can be integrated within digital mammography units and hospital cloud-based network infrastructure for fast assessment while maintaining high accuracy. In addition, inclusion of explainable AI (XAI) components will enable the clinicians to see feature importance and model rationale, building trust and transparency in automated predictions. These advances represent a significant advancement towards clinically adaptive, real-time DS algorithms that may aid human experts in earlier diagnosis of breast cancer.

### 3.6 Ethical Aspects of AI and ML-based Health Care Services

The use of AI for healthcare, such as breast cancer detection, has come with significant ethical responsibilities related to the privacy and security of patient data and fair algorithmic decision-making. AI solutions use sensitive medical data on huge scales for training and validation, thus ensuring that the confidentiality of data is maintained in accordance with healthcare regulations. Any patient information needs to remain anonymous, secure, and used with the consent of the owner to avoid tampering or unauthorized access. The introduction of XAI creates an opportunity to provide mechanisms for rationalizing model decisions that can be interpreted by clinicians, thereby bringing both accountability and trust. Furthermore, cooperation among data scientists, ethicists, and clinicians is required for creating ethical governance models that can lead a responsible developing and deploying of AI systems. These ethical considerations must be addressed to avoid the situation where AI-driven diagnostic models are accurate but biased, insecure, and inconsistent with patient-centered approaches.

## CONCLUSION AND FUTURE WORK

The proposed hybrid framework integrates MDO-based segmentation, U-Net feature extraction, SRO-driven feature optimization, and DSS-CNN classification to enhance early breast cancer detection from mammograms. MDO effectively delineates tumor regions across multiple tissue areas, while SRO refines the feature space by selecting the most relevant descriptors. The combined pipeline significantly improves diagnostic accuracy, reduces dimensionality, and offers a reliable approach for robust and early breast cancer identification. The publicly available CBIS-DDSM dataset is used to validate the effectiveness. From the results, analyzed that the proposed DSS-CNN model achieves maximum performance such as 98.598% of accuracy, 97.343% of precision, 97.515% of sensitivity, 97.689% of specificity, 95.898% of MCC and 97.989% of mAP which is 0.05%, 0.036%, 0.041%, 0.035%, 0.09% and 0.061% efficient than the existing state of art models. Future work will focus on evaluating the proposed pipeline across multiple, diverse mammography datasets to assess its adaptability. Additionally, lightweight optimization techniques and hardware accelerators (*e.g*., FPGA or edge AI deployment) will be explored to enable faster inference. The integration of multimodal medical data, such as ultrasound or MRI, and explainable AI techniques will also be investigated to enhance interpretability and clinical trust in diagnostic outcomes.

## Figures and Tables

**Fig. (3):**
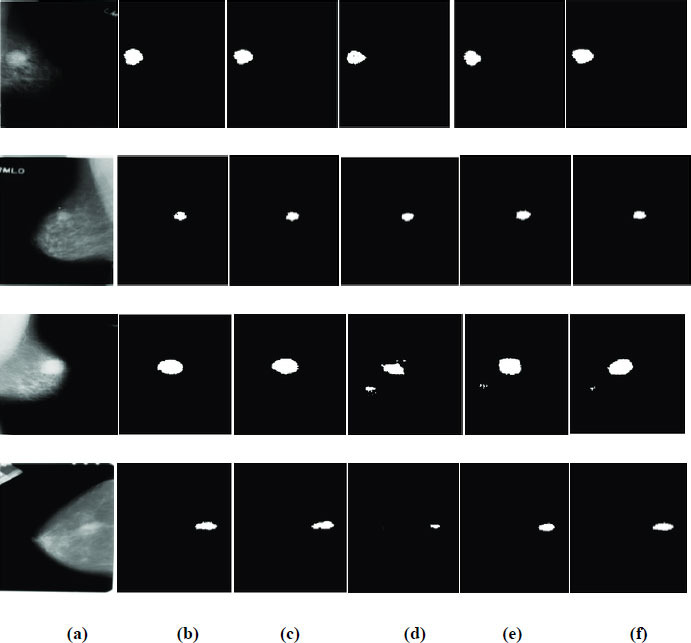
Results of segmentation (**a**) Samples of the CBIS-DDSM dataset (**b**) Mask image (**c**) PSO algorithm (**d**) ACO algorithm (**e**) WSO algorithm (**f**) Proposed MDO algorithm.

**Table 1:** Summary of research gaps from breast cancer detection and classification.

**Ref.**	**Feature Extraction**	**Classification**	**Dataset**	**Findings**	**Research Gaps**
[21]	AlexNet, ResNet, and MobileNetV2	Modified high-boosting	Mini-DDSM, BUSI	Accuracy 96.92%	Affected by incidence and not suitable for early detection
[22]	Probabilistic PCA	TCNN + MMFLO	OMI-GE	Accuracy 96.56%	Faces huge false-negative results and a lower detection rate
[23]	Statistical significance analysis	Support vector machine (SVM)	CBIS-DDSM	Accuracy 96.44%	Interpretability, data reliance, and computational complexity
[24]	Logistic regression	Hybrid deep recurrent network	BUSI and BUS2	Accuracy 89.56%	Leads to a redundant biopsy that increases the false-negative value
[25]	LTP, FLBP, median binary patterns	Q-SpinalNet	Mini-DDSM	Accuracy 90.9%	Making it difficult to accurately detect tumors with different shapes
[26]	VGGNet and SE-ResNet152	CNN+LSTM and CNN+SVM,	OMI-GE	Sensitivity 99%	Extracted features often suffer from poor robustness
[27]	Fuzzy C means clustering	SVM and K-NN	CBIS-DDSM	Accuracy 96.52%	Feature extraction using a backbone network is insufficient
[28]	CLAHE and AGLCM	WA-BTLBO with XGBoost	MIAS	Accuracy 89.95%	Affected by the detection rate, which limits the false rejection rate
[29]	SWChOA	ZFNet-WChOA-ELM	CBIS-DDSM	Accuracy 95.21%	They did not address the data dimensionality issues
[30]	InceptionResNetV2 and VGG16	MobileNetV2	CBIS-DDSM	Accuracy 96.42%	Due to physiological features, accurate detection of lesions is still difficult

**Table 2:** Description of CBIS-DDSM dataset.

Number of samples	Classes		Total
	Benign	Malignant	
Training	1425	1494	2919
Validation	895	564	1459
Testing	225	262	487
Total	2545	2320	4865

**Table 3:** Comparative analysis of different segmentation algorithms.

**Segmentation Algorithm**	**Values in %**			
	**Accuracy**	**Dice**	**Jaccard**	**MCC**
PSO	91.902	90.712	90.403	90.200
ACO	93.504	92.314	92.005	91.802
WSO	95.106	93.916	93.607	93.404
MDO	98.758	97.568	97.259	97.056

**Table 4:** Comparative analysis of proposed and baseline models for breast cancer detection and classification for the CBIS-DDSM dataset with varying epochs.

**Models**	**Number of Epochs**									
	**100**	**200**	**300**	**400**	**500**	**100**	**200**	**300**	**400**	**500**
	Accuracy (%)					Precision (%)				
LR	75.293	75.512	75.824	76.066	76.136	84.259	84.385	84.635	85.048	85.388
DT	77.647	77.866	78.178	78.420	78.490	85.584	85.710	85.960	86.373	86.713
RF	80.001	80.220	80.532	80.774	80.844	86.909	87.035	87.285	87.698	88.038
KNN	82.355	82.574	82.886	83.128	83.198	88.234	88.360	88.610	89.023	89.363
SVM	84.709	84.928	85.240	85.482	85.552	89.559	89.685	89.935	90.348	90.688
CNN	87.063	87.282	87.594	87.836	87.906	90.884	91.010	91.260	91.673	92.013
RNN	89.417	89.636	89.948	90.190	90.260	92.209	92.335	92.585	92.998	93.338
LSTM	91.771	91.990	92.302	92.544	92.614	93.534	93.660	93.910	94.323	94.663
DSS-CNN	98.125	98.344	98.656	98.898	98.968	96.859	96.985	97.235	97.648	97.988
	Recall (%)					F-measure (%)				
LR	81.872	81.941	82.363	82.672	82.805	86.473	86.488	86.886	87.281	87.548
DT	83.520	83.589	84.011	84.320	84.453	87.581	87.597	87.994	88.389	88.657
RF	85.168	85.237	85.659	85.968	86.101	88.693	88.710	89.106	89.501	89.769
KNN	86.816	86.885	87.307	87.616	87.749	89.808	89.826	90.221	90.616	90.884
SVM	88.464	88.533	88.955	89.264	89.397	90.926	90.945	91.339	91.734	92.002
CNN	90.112	90.181	90.603	90.912	91.045	92.047	92.067	92.460	92.855	93.123
RNN	91.760	91.829	92.251	92.560	92.693	93.171	93.191	93.584	93.979	94.247
LSTM	93.408	93.477	93.899	94.208	94.341	94.297	94.318	94.710	95.105	95.373
DSS-CNN	97.056	97.125	97.547	97.856	97.989	96.426	96.449	96.839	97.234	97.501

**Table 5:** Comparative analysis of proposed and existing state-of-the-art models for breast cancer detection and classification for the CBIS-DDSM dataset.

**Models**	**Values in %**							
	**Accuracy**	**Precision**	**Sensitivity**	**Specificity**	**MCC**	**mAP**	**FPR**	**FNR**
O-YOLOv5s	90.501	90.902	90.012	91.023	81.025	88.745	0.092	0.102
M-YOLOv5s	96.125	96.935	95.365	97.148	92.029	95.206	0.056	0.033
O-YOLOv5m	90.004	89.214	91.454	89.647	80.025	87.209	0.111	0.092
M-YOLOv5m	95.503	96.903	94.015	97.354	91.041	95.347	0.065	0.034
O-YOLOv5l	91.525	91.082	92.258	91.342	83.045	88.902	0.091	0.085
M-YOLOv5l	96.345	96.312	95.647	97.698	92.028	95.207	0.056	0.032
O-YOLOv5x	92.514	92.087	93.025	92.356	85.003	89.203	0.082	0.078
M-YOLOv5x	96.503	97.002	96.047	97.429	93.609	96.007	0.041	0.039
DSS-CNN	98.598	97.343	97.515	97.689	95.898	97.989	0.032	0.021

## Data Availability

• The Authors Used the publicly available CBIS-DDSM Breast Cancer image dataset for the experimentation • https://www.kaggle.com/datasets/awsaf49/ cbis-ddsm-breast-cancer-image-dataset

## LIST OF ABBREVIATIONS

CBIS-DDSM: Curated Breast Imaging Subset of the Digital Database for Screening Mammography
ROI: Region of Interest
MDO: Modified Dingo Optimization
SRO: Search and Rescue Optimization
DSS-CNN: Dual Stage Spiking Convolutional Neural Network
